# A clinical randomized controlled trial: moxibustion at Laogong interval with *Panax notoginseng* promoted the maturation of arteriovenous fistulae

**DOI:** 10.1186/s13020-022-00604-9

**Published:** 2022-04-20

**Authors:** Yurou Chen, Lin Xu, Di Huang, Dongping Chen, Feng Wu, Luobing Wang, Jie Zhou, Tianying Lan, Xuehua Qin, Chaoyang Ye

**Affiliations:** 1 Department of Nephrology, Shuguang Hospital Affiliated to Shanghai University of Traditional Chinese Medicine, No. 528, Zhangheng Road, Pudong New District, Shanghai, 201200 China; 2 TCM Institute of Kidney Disease, Shanghai University of Traditional Chinese Medicine, Shanghai, China; 3 Key Laboratory of Liver and Kidney Diseases (Shanghai University of Traditional Chinese Medicine), Ministry of Education, Shanghai, China; 4 Shanghai Key Laboratory of Traditional Chinese Clinical Medicine (20DZ2272200), Shanghai, China

**Keywords:** Moxibustion, *Panax notoginseng*, Laogong(PC8), Arteriovenous fistula, Vascular access

## Abstract

**Background:**

We aim to study the clinical effect of moxibustion at Laogong interval with *Panax notoginseng* on the short-term maturation and long-term patency of arteriovenous fistula.

**Methods:**

Seventy-four pre-dialysis uremic patients who received distal forearm radial-cephalic fistula creations were enrolled in this study and randomly assigned to the control group and experimental group. After arteriovenous fistula creations, the control group underwent handgrip exercise, and the experimental group received moxibustion at Laogong acupoint interval with *Panax notoginseng*. Both groups received a 12-week treatment and were followed up for 24 weeks in all at the following time points: before creations and 2, 4, 8, 12, 24 weeks after creations. The diameter of anastomosis, the diameter and outflow of draining-veins 5 cm above anastomosis, the diameter and outflow of brachial arteries evaluated the maturation and patency of arteriovenous fistula. Enzyme linked immunosorbent assay determined serum levels of endothelin and nitric oxide.

**Results:**

The maturity rate in the experimental group was significantly higher than that in the control group at 4 weeks after arteriovenous fistula creations (P = 0.048). The diameter of anastomosis, the diameter of draining veins, and the blood flow of draining veins increased in both groups during the whole 24 weeks. The diameter and blood flow of brachial arteries ascended in both groups during the previous 12 weeks. Compared with the control group, moxibustion at Laogong interval with *Panax notoginseng* significantly improved the value of the diameter of draining-veins (P = 0.016), the blood flow of draining-veins (P = 0.015), the diameter of brachial arteries (P < 0.001), and the blood flow of brachial arteries (P = 0. 012) at 2 weeks, and enhanced the blood flow of draining-veins (P = 0.029) and brachial arteries (P < 0.001) at 12 weeks. Serum levels of endothelin were significantly lower (P = 0.047), and serum levels of nitric oxide were markedly higher (P < 0.001) in the experimental group than that in the control group at 2 weeks after creations.

**Conclusions:**

Moxibustion at Laogong interval with *Panax notoginseng* was non-invasive and promoted the maturation of arteriovenous fistula at 4 weeks after creations. However, its long-term beneficial effect on patency at 24 weeks after creations was not significant.

*Trial registration* Chinese Clinical Trial Registry, No. ChiCTR1900024042. Registered, http://www.chictr.org.cn/index.aspx

## Background

Arteriovenous fistula (AVF) is a commonly used vascular pathways [1, 2] when venous segments need to be expanded and thickened to mature for usage. After AVF creation, the hemodynamic changes such as the sudden increasing blood flow of draining veins (BFV), the velocity variation of distributed and turned blood flow at anastomotic stoma, and the non-laminar and disordered blood flow in vessels may induce adaptive alteration of AVF, particularly when combined with other risk factors, for example, uremic toxin accumulation, systemic inflammatory responses, oxidative stress, and vascular lesions. Currently, the short-term maturation and the long-term patency rate are still unsatisfied in clinical practice. The failure rate of maturation in 5 months after AVF creation is as high as 60% [3]. Although there have been many strategies to promote the maturation and long-term patency of AVF in clinics, most of them are not effective [4]. Vessel stenosis due to neointimal hyperplasia and blood flow insufficient for dialysis are two critical causes for the immature and dysfunction of AVF [5]. The immature and dysfunction of AVF is associated with weakening pulse, shudder and murmur at venous segments and swelling hands sometimes combined with pain and finger paralysis, which is like "Hand arthralgia," a traditional Chinese medicine (TCM) disease. "Hand arthralgia" presents with painful hands or arms associated with “Qi” stagnation and blood stasis due to vessel injury. The TCM therapeutic principle "Huoxue Huayu" promotes blood circulation and eliminates blood stasis, is widely applied in China to cure "Hand arthralgia." *Panax notoginseng* is a kind of "Huoxue Huayu" medicine. Pharmacological studies have showed that *Panax notoginseng* prevents thrombosis formation by inhibiting the production of thromboxane A2 and releasing calcium 5-hydroxytryptamine [6]. Moxibustion is one of the most effective "Huoxue Huayu" therapies in China. Modern studies have suggested that thermal stimulation of moxibustion improves microcirculation [7] and increases the blood flow of local capillaries, acrovascular [8], and visceral vessels [9]. Moxibustion may benefit vasodilation due to the release of norepinephrine [10] and vasoactive intestinal peptide [11] after nerve-muscle sympathetic excitation. Laogong acupoint is one of the primary acupoints for treating "Hand arthralgia," according to TCM experience. Based on mentioned above, we hypothesize that moxibustion at Laogong interval with *Panax notoginseng* (MLPN) would benefit AVF. In this study, we observe the effect of MLPN on the short-term maturation and long-term patency of AVF.

## Methods

### Study design and participants

**Fig. 1:**
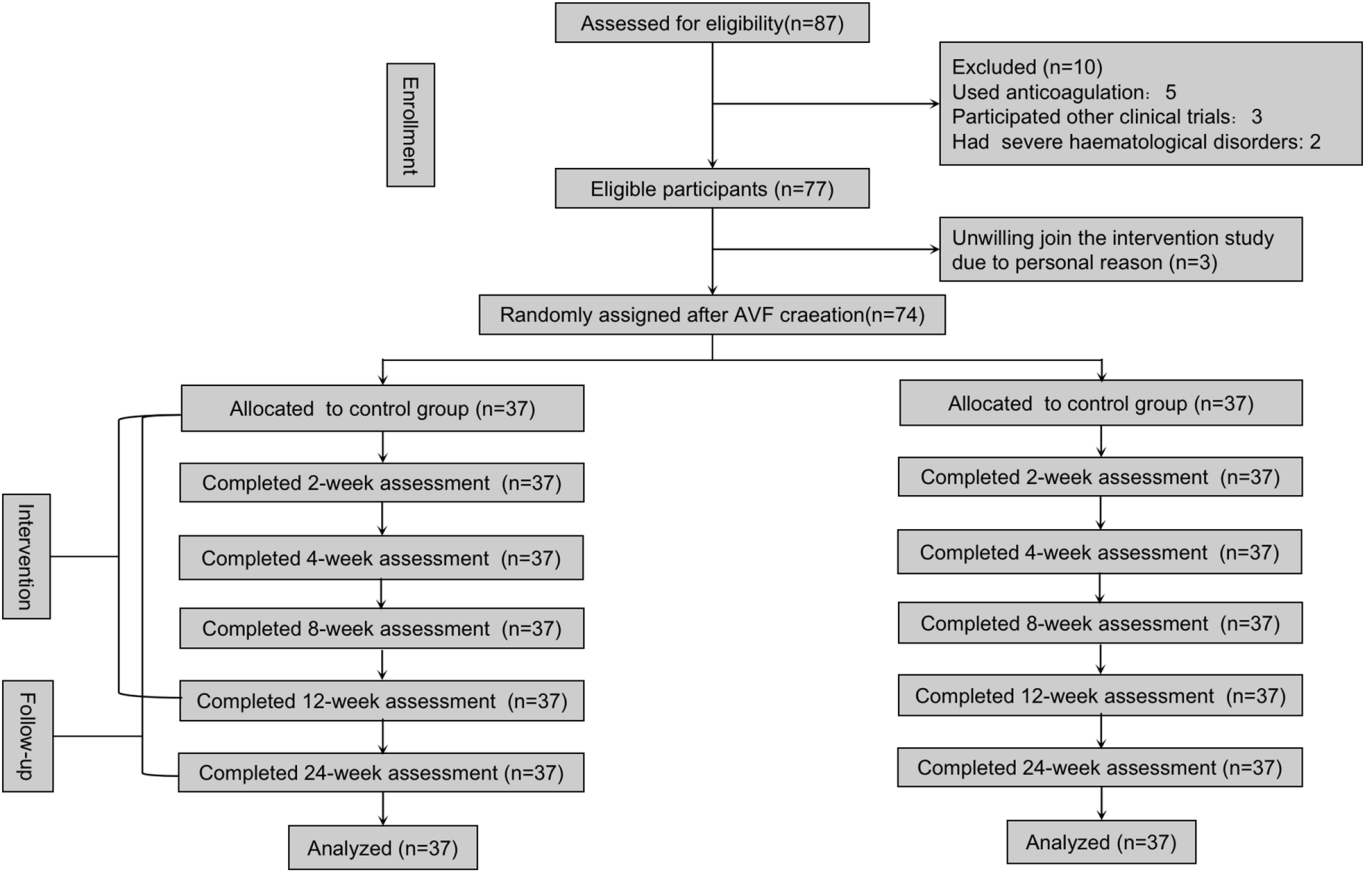
The flow chart of the trial

The study was a prospective, single-center, randomized controlled trial with a 24-week follow-up (Fig. 1).

This study was carried out between June 2019 and November 2020 in the Department of nephrology, Shuguang Hospital Affiliated to Shanghai University of TCM, China. Seventy-four predialysis uremic patients, aged 18 to 80 years old with signed informed consents, underwent radial-cephalic AVF (RC-AVF) creations were enrolled. The exclusion criteria included the following factors:

- without chronic renal failure (CRF);
- with severe cerebrovascular, liver diseases;
- after kidney transplantation;
- with acute renal failure;
- pregnant or lactating women;
- hypersensitive to pseudo-ginseng or moxibustion;
- with mental disease;
- participation in other clinical trials in the meantime;
- using other antithrombotic medicine within 3 months;
- unwilling to participate in this trial.

A random counting Excel table was in use. Before the intervention, patients were randomly allocated to the experimental group or the control group (1: 1 ratio).

### Drug preparation

*Panax notoginseng* powder was provided by Instant Herb of Shuguang Hospital Affiliated to Shanghai University of Traditional Chinese Medicine, mixed with millet wine (2:1 ratio) and made as circle cakes with 2–3 cm diameters. 5–10 holes were burrowed into each herbal cake by 0.45 mm needle [12, 13]. Moxa was bought from Hanyi Moxa of Henan Nanyang Limited Company and kneaded to be cones with 1.2 cm in diameter in the bottom and 1.5 cm in height.

### Interventions

**Fig. 2:**
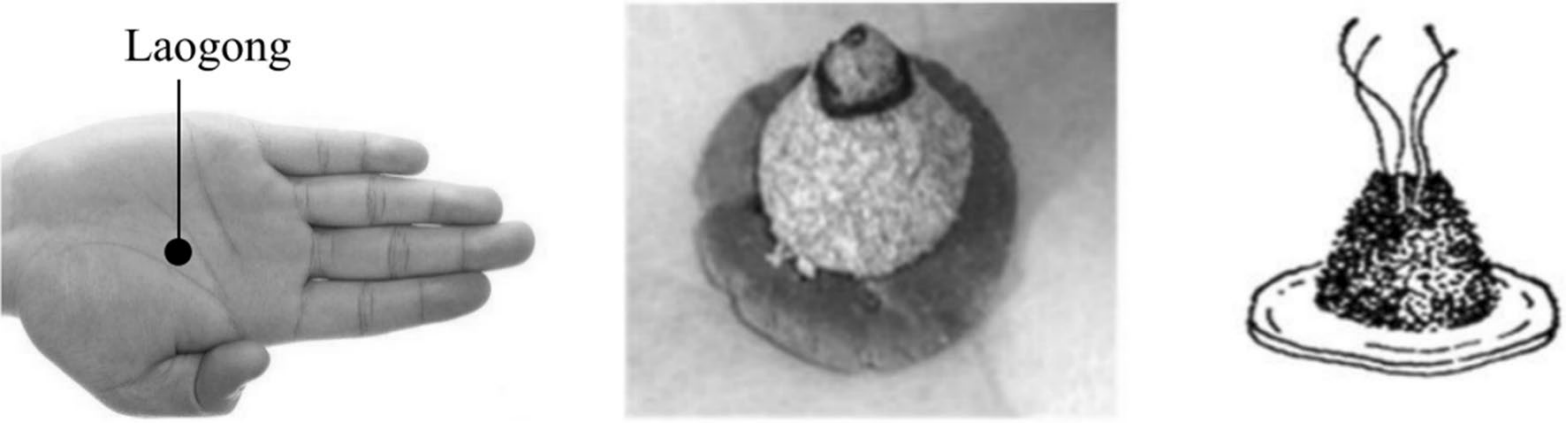
Moxibustion separated by PNC at Laogong. PNC: *Panax notoginseng* cakes

Both groups underwent RC-AVF creations in the distal forearm by two experienced surgeons who had been engaged in vascular-access surgery for over 10 years. After AVF creations, participants in the experimental group received moxibustion interval with *Panax notoginseng* cakes (PNC) at Laogong (Fig. 2). Laogong is located between the second and third metacarpals in the palm, which could be touched by the middle finger when making a fist. Moxa cones were put on PNC and kindled from the tips. A total of three moxa cones burned up for 30-45 min per session. Moxibustion was performed daily at the first week after creations and three times per week from the second week. Participants in the control group were instructed to squeeze rubber balls for 15 min twice a day and advised to perform the exercise per day [14]. Both interventions lasted for 12 weeks.

Both groups received integrated therapy on CRF. When such severe uremic symptoms as high potassium, severe metabolic acidosis, and acute heart failure appeared, the participants began to dialysis according to the objective laboratory parameters and the subjective judgment of doctors.

### Outcome measures

The primary outcomes of this study were the diameter and outflow of draining-veins 5 cm above anastomosis, the maturity rate of AVF at 4 weeks and the patency rate at 24 weeks after creations. AVF maturity was assessed by two experienced nephrologists skilled in AVF examination. Clinical AVF maturation was defined as an easily palpable vein by physical examination, including the arm-raising and augmentation tests, with a straight-superficial segment, length of more than 10 cm, sufficient diameter, and good palpable thrill. Ultrasound criteria for AVF maturation were defined as diameter of draining vein (DV) ≥ 5 mm, distance of skin-vein ≤ 6 mm, and BFV ≥ 500 ml/min [1].

The secondary outcomes were the diameter of anastomosis (DA), the diameter of brachial artery (DBA), the blood flow of brachial artery (BFBA), and the incidence of adverse events such as thrombosis, hemorrhage, aneurysm and heart failure for 24 weeks. DBA and BFBA were measured at 2 cm above antecubital fossa in the inner arm. A color Doppler ultrasound scanner (MyLab30CV, 104730010) equipped with a linear probe was applied to measure vessels' blood flow and diameter by two trained nephrologists, strictly following the measurement guidelines [15]. Ultrasonographic datum were collected at six time points: before AVF creations (baseline T0) and 2 (T1), 4 (T2), 8 (T3), 12 (T4), 24 (T5) weeks after the creation. Serum samples were collected at T0, T4, T5 and stored at − 80 °C for further enzyme linked immunosorbent assay tests (endothelin (ET) kits: Jianglai Biology Company, JL10968, 96T, nitric oxide (NO) kits: Shanghai Hailing Biotechnology Co. Ltd, HLE11572, 96T).

### Statistical methods

The sample size calculation was performed as described by our previous study, in which the maturity rate of MLPN at 4 weeks after AVF creations was 76%. With a power of 0.8 and a type I error rate of 5%, the estimated sample size was 37 patients in each group calculated by the formula n = $\frac{2\overline{\mathrm{p} }\overline{\mathrm{q} }{({\mathrm{Z}}_{\mathrm{\alpha }}+{\mathrm{Z}}_{\upbeta })}^{2}}{{(\mathrm{p}1-\mathrm{p}2)}^{2}}$. All datum were analyzed by SPSS software (version 22.0). The statistical variables in this study were in normal distribution and described as $\overline{x}$ ± SD. An independent t-test was used for comparing continuous variables between the MLPN group and the control group. The categorical variables were tested by the chi-square test or Fisher's exact test. The variations in diameter and blood flow in each group at six-time points were analyzed by one-way ANOVA test. P-value < 0.05 was considered statistically significant.

## Results

### Participants

Table 1 showed the baseline characteristics of the participants in this trial. There was no significant difference in baseline parameters between the MLPN group and the control group.

**Table 1:** Baseline characteristics of the participants in MLPN group and control group

	MLPN group (n = 37)	Control group (n = 37)	t	P
Age, years, $\overline{x}$ ± SD	61.57 ± 10.02	56.59 ± 12.76	1.864	0.066
Gender, male/female, n	26/11	26/11	0	1
Primary disease, n (%)			3.5	0.316
Chronic glomerulonephritis	8 (21.6)	12 (32.4)		
Hypertensive nephropathy	9 (24.3)	8 (21.6)		
Diabetic nephropathy	9 (24.3)	12 (32.4)		
Others	11 (29.7)	5 (13.5)		

MLPN: moxibustion at Laogong interval with *panax notoginseng*

### Primary outcome

Table 2 displayed the short-term maturity rate and long-term patency rate of AVF. At 4 weeks after creations, there were 34 cases in the MLPN group, and 26 cases in the control group met the mature criteria. The maturity rate of the MLPN group was 91.89%, and it was higher than that in control one (70.3%, p = 0.048). At 24 weeks after AVF creations, there were both 28 effective fistulas, and the patency rates were 75.68%.

**Table 2:** 4-week maturity rate and 24-week patency rate of AVF secondary outcomes

	MLPN group (n = 37)	Control group (n = 37)	P
Maturity rate,% (n)	91.89 (34)	70.27 (26)	0.048
Patency rate,% (n)	75.68 (28)	75.68 (28)	1.000

AVF: arteriovenous fistula; MLPN: moxibustion at Laogong interval with *panax notoginseng*

### Secondary outcomes

**Fig. 3:**
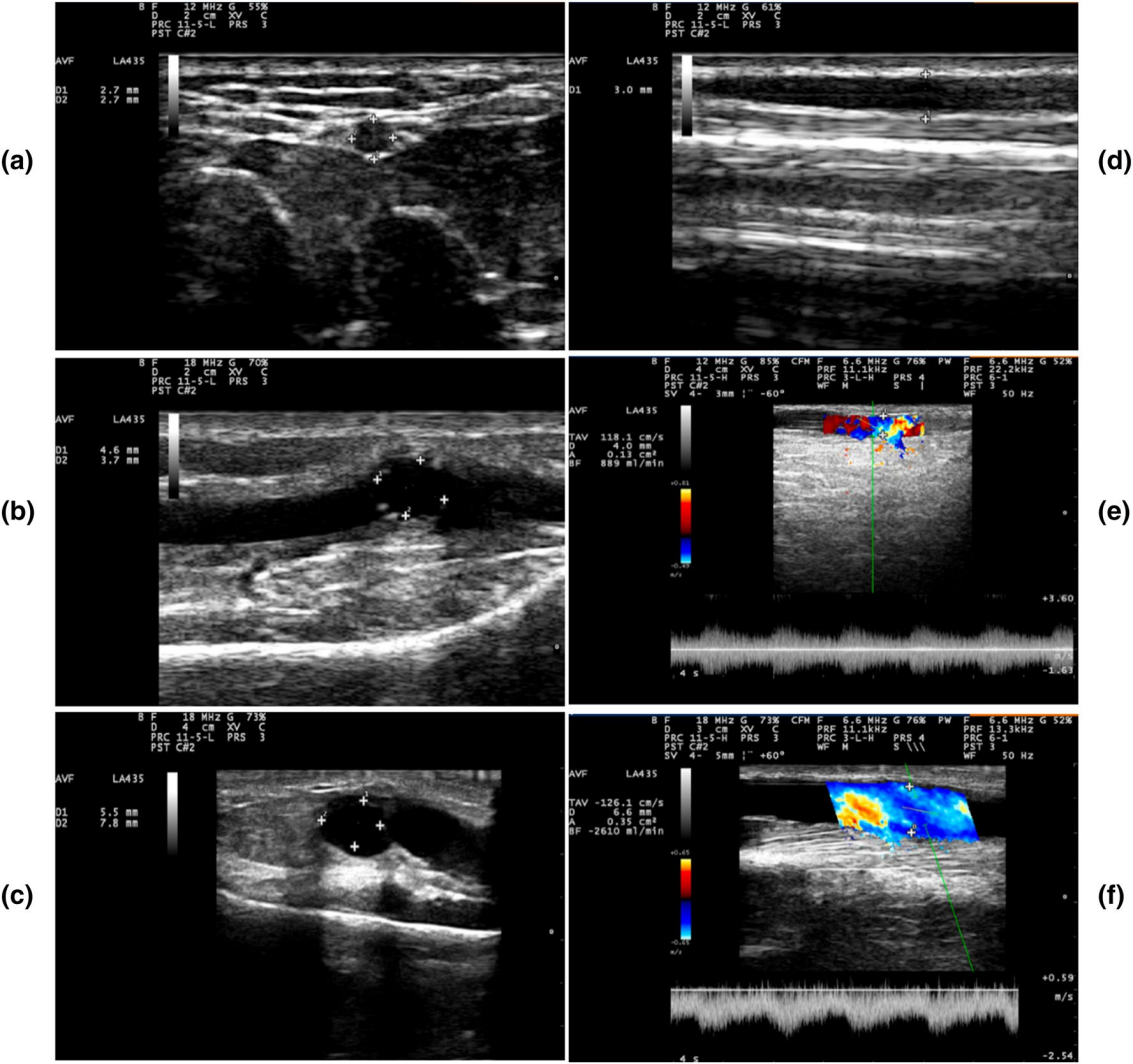
The diameter and the blood flow of vessels in MLPN group under ultrasound examination: **a** DA (2.7*2.7 mm) at T0, **b** DA (4.6*3.7 mm) at T2, **c** DA (5.5*7.8 mm) at T5, **d** DV (3.0 mm) at T0, **e** DV (4.0 mm) and BFV (889 ml/min) at T0, **f** DV (6.6 mm) and BFV (2610 ml/min) at T5. MLPN: moxibustion at Laogong interval with *Panax notoginseng*; DA: diameter of anastomosis; DV: diameter of draining-veins; BFV: blood flow of draining-veins

Table 3 displayed the dynamic changes in DA, DV, and BFV over time. Both DA and DV in the MLPN group ascended in the whole 24-week follow-up (Fig. 3). During the 12-week intervention, BFV in MLPN group continued increasing, while reversed to decrease after the intervention. One statistical difference between two groups appeared at T1. Compared to those in the control group, DV (p = 0.016) and BFV (p = 0.015) in the MLPN group was significantly increased. Another at T4, BFV in the MLPN was higher than in the control group (p = 0.029). Taken together, MLPN improved DV and BFV compared with single handgrip exercise.

**Table 3:** DA, DV, and BFV of AVF in six-time points ($\overline{x}$ ± SD)

		MLPN group (n = 37)	Control group (n = 37)	t	P
T0	DA, mm	2.59 ± 0.99	2.51 ± 0.76	0.425	0.672
	DV, mm	2.69 ± 0.79	2.76 ± 0.86	0.387	0.700
T1	DA, mm	3.60 ± 0.96^***^	3.41 ± 0.76^***^	0.912	0.375
	DV, mm	4.12 ± 0.98^***^	3.59 ± 0.89^***^	2.463	0.016
	BFV, ml/min	771.9 ± 374.42	571.51 ± 305.38	2.503	0.015
T2	DA, mm	3.97 ± 0.96^***^	3.91 ± 0.88^***^	0.266	0.791
	DV, mm	4.44 ± 0.82^***^	3.99 ± 1.22^***^	1.831	0.071
	BFV, ml/min	935.49 ± 444.01	752.97 ± 395.38^#^	1.867	0.066
T3	DA, mm	4.26 ± 1.03^***^	4.08 ± 0.94^***^	0.776	0.440
	DV, mm	4.86 ± 1.00^***^	4.37 ± 1.28^***^	1.849	0.069
	BFV, ml/min	996.30 ± 456.86^#^	811.72 ± 424.29^##^	1.801	0.076
T4	DA, mm	4.65 ± 1.03^***^	4.23 ± 1.00^***^	1.780	0.079
	DV, mm	5.03 ± 1.07^***^	4.70 ± 1.47^***^	1.138	0.259
	BFV, ml/min	1183.87 ± 739.26^##^	864 ± 467.01^##^	2.232	0.029
T5	DA, mm	4.72 ± 1.26^***^	5.07 ± 1.14^***^	0.370	0.712
	DV, mm	5.13 ± 1.08^***^	4.90 ± 1.51^***^	0.706	0.483
	BFV, ml/min	1078.10 ± 590.04^##^	957.75 ± 648.43^##^	0.780	0.438

^***^Significant different compared to T0 (P < 0.001)

^#^ Significant different compared to T1 (P < 0.05), ^##^Significant different compared to T1 (P < 0.01)

AVF: arteriovenous fistula; MLPN: moxibustion at Laogong interval with *Panax notoginseng*; DA: diameter of anastomosis; DV: diameter of draining veins; BFV: blood flow of draining veins

**Fig. 4:**
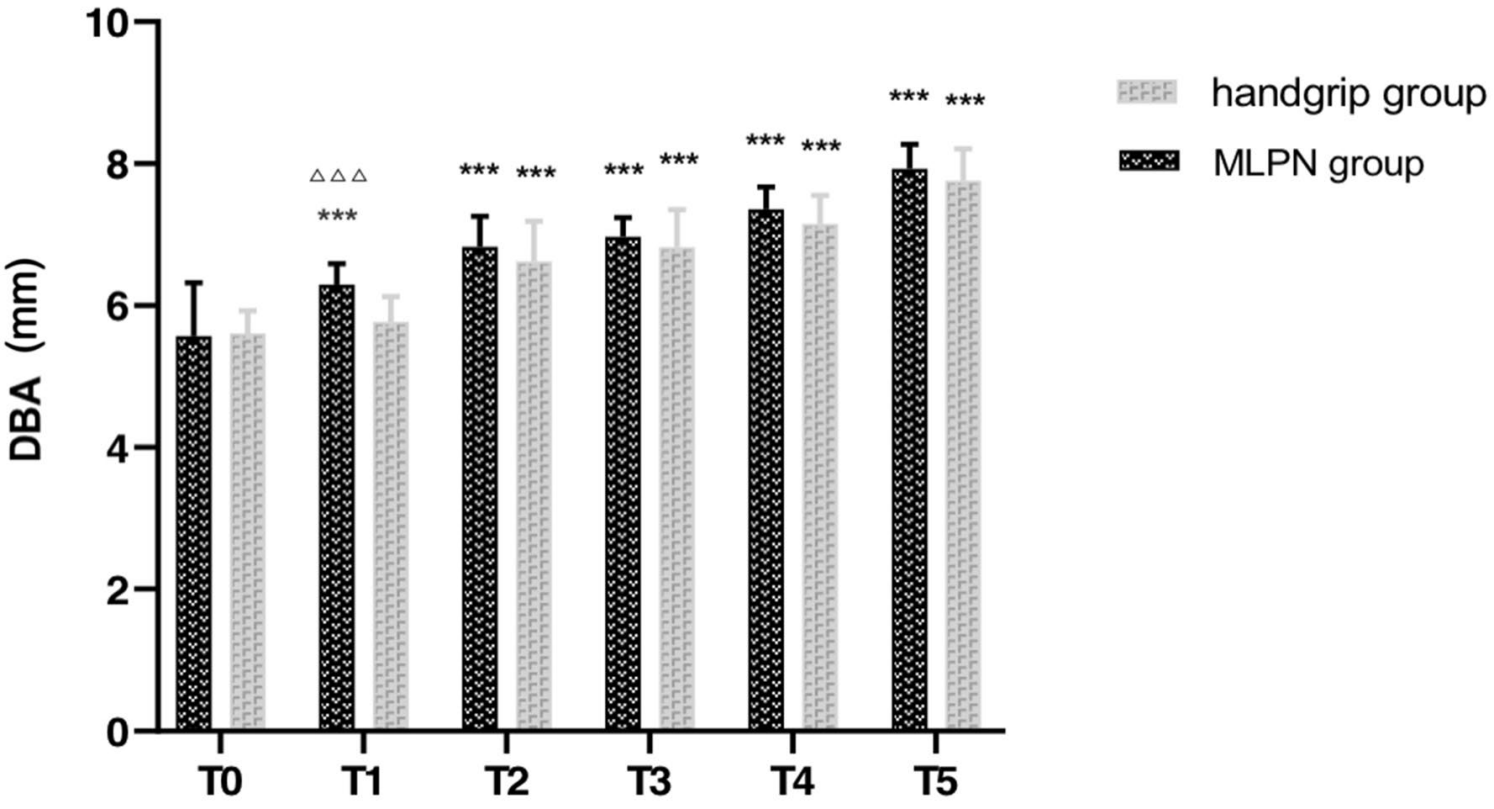
DBA of two groups at six-time points. ***Significant changes compared to T0 (P < 0.001), ^△△△^Significant differences between two groups (P < 0.001). DBA: diameter of brachial arteries

**Fig. 5:**
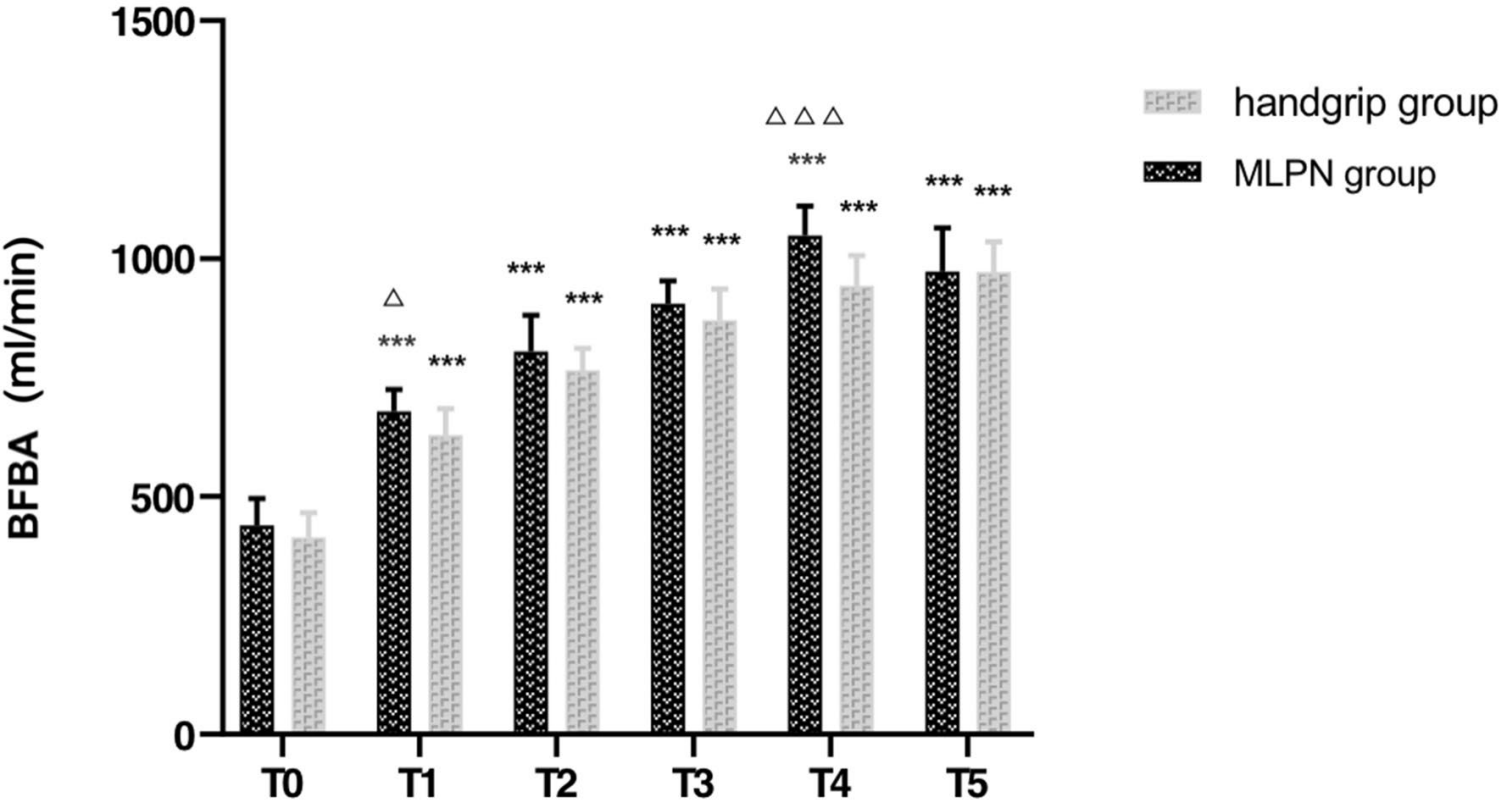
BFBA of two groups at six-time points. ***Significant changes compared to T0 (P < 0.001), ^△^Significant differences between two groups (P < 0.05), ^△△△^Significant differences between two groups (P < 0.001). BFBA: blood flow of brachial arteries

Table 4 presented the changes in DBA and BFBA. The same as DV and BFV, DBA increased in the whole follow-up and BFBA from the previous 12 weeks after AVF creations. DBA and BFBA in the MLPN group significantly improved since T1 (both p < 0.001). There was no statistical difference in both DBA and BFBA between the two groups at T0, while the value of DBA (Fig. 4) and BFBA (Fig. 5) were significantly higher in the MLPN group than those in the control group at T1 (p < 0.001, p = 0.012). BFBA further increased in the MLPN group at T4 (p < 0.001). Together, MLPN improved DBA and BFBA in contrary to the single handgrip exercise in the short term.

**Table 4:** DBA and BFBA in six-time points ($\overline{x}$ ± SD)

		MLPN group (n = 15)	Control group (n = 15)	t	P
T0	DBA, mm	5.57 ± 0.75	5.61 ± 0.32	0.160	0.874
	BFBA, ml/min	439.13 ± 56.57	413.93 ± 51.32	1.278	0.212
T1	DBA, mm	6.30 ± 0.29^***^	5.77 ± 0.36^***^	4.391	0.000
	BFBA, ml/min	678.67 ± 45.31^***^	629.20 ± 54.99^***^	2.689	0.012
T2	DBA, mm	6.83 ± 0.43^***^	6.62 ± 0.57^***^	1.192	0.243
	BFBA, ml/min	805.13 ± 75.33^***^	764.20 ± 47.43^***^	1.781	0.086
T3	DBA, mm	6.97 ± 0.27^***^	6.82 ± 0.53^***^	0.967	0.342
	BFBA, ml/min	904.87 ± 48.15^***^	870.27 ± 65.42^***^	1.650	0.110
T4	DBA, mm	7.36 ± 0.31^***^	7.15 ± 0.40^***^	1.600	0.121
	BFBA, ml/min	1048.70 ± 61.62^***^	942.24 ± 63.79^***^	4.651	0.000
T5	DBA, mm	7.93 ± 0.34^***^	7.76 ± 0.45^***^	1.162	0.255
	BFBA, ml/min	972.84 ± 91.38^***^	971.89 ± 63.73^***^	0.033	0.974

^***^Significant changes compared to T0 (P < 0.001)

MLPN: moxibustion at Laogong interval with *Panax notoginseng*; DBA: diameter of arteria brachialis; BFBA: blood flow of arteria brachialis

Table 5 showed the changes in the serum levels of ET and NO. At T0, there was no significance between these two groups. The serum level of ET had decreased since T1 in the control group, which negatively correlated with NO level (p < 0.001). The serum level of ET in the MLPN group was lower than that in the control group (p = 0.047), while the serum level of NO in the MLPN group was higher than that in the control group (p < 0.001) at T1. This datum implied that MLPN enhanced serum levels of NO and decreased serum levels of ET.

**Table 5:** Serum levels of ET and NO at six-time points ($\overline{x}$ ± SD) (μml/l)

	MLPN group (n = 37)		Control group (n = 37)		P	
	ET	NO	ET	NO	P1	P2
T0	5.51 ± 0.25	29.21 ± 2.67	5.45 ± 0.32	28.92 ± 3.85	0.428	0.701
T1	5.16 ± 0.44^***^	37.78 ± 2.75^***^	5.37 ± 0.45	29.43 ± 3.55	0.047	0.000
T2	5.10 ± 0.60^***^	39.13 ± 3.24^***^	5.19 ± 0.27^**^	38.57 ± 3.49^***^	0.456	0.473
T3	4.92 ± 0.30^***^	39.71 ± 3.05^***^	4.99 ± 0.35^***^	39.18 ± 3.30^***^	0.346	0.473
T4	4.87 ± 0.27^***^	41.03 ± 3.71^***^	4.92 ± 0.36^***^	40.87 ± 3.53^***^	0.539	0.848
T5	4.80 ± 0.34^***^	40.92 ± 3.28^***^	4.84 ± 0.59^***^	40.04 ± 2.88^***^	0.670	0.222

^**^Significant changes compared to T0 (P < 0.01), ***Significant changes compared to T0 (P < 0.001)

MLPN: moxibustion at Laogong interval with *panax notoginseng*; ET: endothelin; NO: nitric oxide

## Discussion

Our study compared the effect of MLPN with that of handgrip exercise on the short-term maturation and long-term patency of AVF. Compared with that in the handgrip group, a significantly increased value in DV, DBA, BFV, and BFBA were observed in the MLPN group at 2 weeks after AVF creations, implying that MLPN arterialized and expanded the draining veins and brachial arteries to accommodate the larger volume of blood flow in the short term. However, although the vascular diameter and the blood flow in the MLPN group were remarkedly higher than in the handgrip group at 2 weeks, the numbers of mature fistulae of these two groups were similar. Therefore, the maturity rate of the MLPN group was not higher than that of the handgrip group. At 4 weeks after AVF creations, the number of mature fistulae in the MLPN group was larger than that in the handgrip group. As a result, our current study showed that MLPN promoted the maturation at 4 weeks after creations.

We were interested in the reasons why MLPN could promote AVF maturation. First of all, It was confirmed that moxibustion and *Panax notoginseng* speeded up blood circulation and eliminated blood stasis [16, 17]. Plus, we found it might be associated with ET and NO in terms of physiology and pathology. NO and ET is a pair of vasodilator and vasoconstrictor factors, playing an essential role in maintaining vascular tension. NO is a membrane penetrated molecule produced by endothelial cells, which produces endothelial NO synthase that prevents platelet aggregation, leukocyte adhesion, and proliferation of vascular smooth muscle cells [18]. ET is an active substance secreted by endothelial cells, which can promote the proliferation of vascular endothelial cells, vascular smooth muscle cells, and fibroblasts [19]. Combined with the enlarged DV, DBA, BFV and BFBA at 2 weeks after creations, the higher serum levels of ET and the lower serum levels of ET in the MLPN groups indicated that vasodilation of MLPN might closely related to NO increase and ET decrease.

**Fig. 6:**
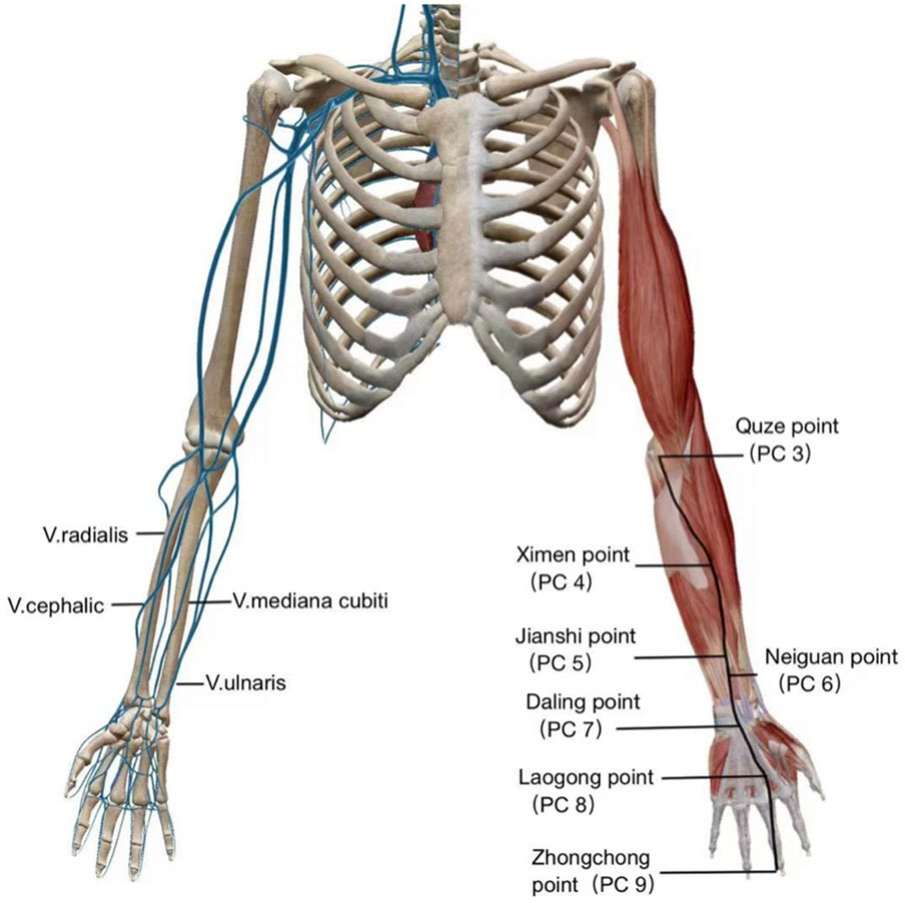
Upper limb vessels and hand Jueyin pericardial meridian

**Fig. 7:**
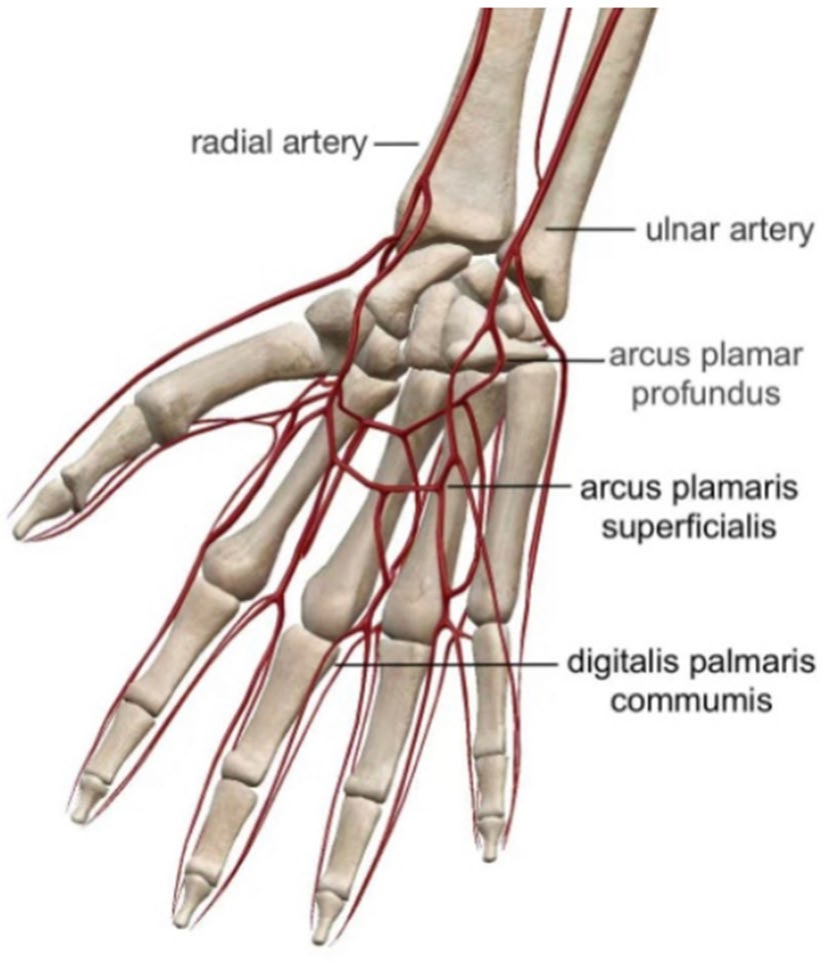
Palmar arches

From the perspective of anatomy, two reasons could account for the improvement. On one side, the therapeutic acupoint—Laogong belongs to the pericardial meridian of hand Jueyin, a channel that coincides with the anatomical location of AVF to a certain extend (Fig. 6). On the other side, the vascular network below Laogong is connected with radial arteries and cephalic veins, the significant parts of AVF (Fig. 7). Based on the meridian theory, "Qi" runs and circulates in the whole meridian [20]. Therefore, even treating Laogong alone would influence the whole hand Jueyin pericardial meridian and the AVF nearby. Laogong as the moxibustion position enhanced the therapeutic effect.

Besides the short-term maturation of AVF, we also compared the effect of MLPN on the long-term patency of AVF with handgrip exercise. Unfortunately, the effect of MLPN on the patency rate of AVF at 24 weeks after operation was not significant. However, the values of BFV and BFBA in the MLPN group were higher than that in the handgrip group during the previous 12-week intervention, implying that continuous MLPN treatment might improve the AVF patency in the long term. The hypothesis needed to be confirmed by extended period of MLPN treatment.

There were several limitations to our study. Firstly, the study was a single-center trial, and most participants came from east of China. Secondly, the scope of samples needed to expand to guarantee the universality of the result. Lastly, measuring conditions should be controlled, such as room temperature, measuring time, and fixed place.

## Conclusions

MLPN improved the maturation of AVF in the short term. Our study suggested that CRF patients who needed to start hemodialysis as soon as possible might benefit from this new non-invasive, practical therapeutic application.

## Data Availability

The datum that support the findings of this study are available from the corresponding author upon reasonable request.

## Abbreviations

AVF: Arteriovenous fistula
BFV: Blood flow of draining veins
TCM: Traditional Chinese medicine
MLPN: Moxibustion at Laogong interval with *Panax notoginseng*
RC-AVF: Radial-cephalic AVF
CRF: Chronic renal failure
PNC: *Panax notoginseng* Cakes
DV: Diameter of draining vein
DA: Diameter of anastomosis
DBA: Diameter of brachial artery
BFBA: Blood flow of brachial artery
ET: Endothelin
NO: Nitric oxide
